# Fish Models of Induced Osteoporosis

**DOI:** 10.3389/fcell.2021.672424

**Published:** 2021-06-10

**Authors:** Joana T. Rosa, Vincent Laizé, Paulo J. Gavaia, M. Leonor Cancela

**Affiliations:** ^1^Centre of Marine Sciences, University of Algarve, Faro, Portugal; ^2^S2 AQUA - Sustainable and Smart Aquaculture Collaborative Laboratory, Olhão, Portugal; ^3^GreenCoLab - Associação Oceano Verde, Faro, Portugal; ^4^Faculty of Medicine and Biomedical Sciences, University of Algarve, Faro, Portugal; ^5^Algarve Biomedical Center, University of Algarve, Faro, Portugal

**Keywords:** teleosts, fish model, induced osteoporosis, screening, bone anabolic compounds

## Abstract

Osteopenia and osteoporosis are bone disorders characterized by reduced bone mineral density (BMD), altered bone microarchitecture and increased bone fragility. Because of global aging, their incidence is rapidly increasing worldwide and novel treatments that would be more efficient at preventing disease progression and at reducing the risk of bone fractures are needed. Preclinical studies are today a major bottleneck to the collection of new data and the discovery of new drugs, since they are commonly based on rodent *in vivo* systems that are time consuming and expensive, or *in vitro* systems that do not exactly recapitulate the complexity of low BMD disorders. In this regard, teleost fish, in particular zebrafish and medaka, have recently emerged as suitable alternatives to study bone formation and mineralization and to model human bone disorders. In addition to the many technical advantages that allow faster and larger studies, the availability of several fish models that efficiently mimic human osteopenia and osteoporosis phenotypes has stimulated the interest of the academia and industry toward a better understanding of the mechanisms of pathogenesis but also toward the discovery of new bone anabolic or antiresorptive compounds. This mini review recapitulates the *in vivo* teleost fish systems available to study low BMD disorders and highlights their applications and the recent advances in the field.

## Introduction

Osteoporosis (OP) is an age-related metabolic disease characterized by low bone mineral density (BMD), the deterioration of bone architecture and the occurrence of fragility fractures (reviewed in Compston et al., 2019). Its prevalence is increasing worldwide, and it is likely that the steady increase in life expectancy will aggravate this already worrying situation. Current treatments mostly rely on anti-resorptive drugs (Tu et al., 2018) that are also associated with limited long-term efficacy and critical side effects, highlighting the importance to search for new therapeutics. Alternative therapies based on new technologies like mesenchymal stem cells and reverse genetics using sncRNAs and miRNAs (reviewed in De Martinis et al., 2019) are being developed. However, much of the research effort is nowadays concentrated on the discovery of bone anabolic agents that would promote proliferation/differentiation of osteoblasts (Chen M. et al., 2014) or increase bone mineral deposition to counteract BMD reduction. The discovery and development of osteogenic drugs require not only a deep understating of OP molecular players but also high-throughput screening pipelines that can identify compounds in large molecule libraries. Despite metabolic and anatomic differences with humans, ovariectomized rodents have become a reference model for OP research and early stage testing of compounds (Turner, 2001; van‘t Hof, 2011; Sophocleous and Idris, 2014; Komori, 2015), but issues related to costs and low screening throughput have limited their use in particular areas of OP studies. The growing interest in fish species to model human diseases (Schartl, 2014), in particular zebrafish and medaka, and their validation as suitable alternatives to study human skeletal disorders (reviewed in Laizé et al., 2014; Witten et al., 2016; Carnovali et al., 2019a; Tonelli et al., 2020), has contributed to favor their use to address OP pathophysiology (reviewed in Bergen et al., 2019). The development of fish OP models that respond to classical anti-OP drugs gave rise to new tools to accelerate bone research and to test and characterize new drugs for OP treatment. In this review, we will provide an overview of these models, their suitability as systems for phenotype-based screenings and the advantages of using these small non-mammalian vertebrates, as well as their applications, in the study of osteoporosis.

## Suitability of Teleost Fish to Study Osteoporosis

Small teleost fish such as zebrafish and medaka have been increasingly used as models for human skeletal diseases as they hold many anatomic and developmental features of mammalian skeletons (reviewed in Laizé et al., 2014; Witten et al., 2016; Carnovali et al., 2019a; Tonelli et al., 2020). Teleostean and mammalian axial and appendicular skeletons are similar in many aspects: nearly all the bones are matching and genes, cells and mechanisms controlling skeletogenesis, including the early formation of the cartilaginous anlage and its replacement by bone, through endochondral and perichondral ossification, and the dermal ossification processes, are highly conserved (Javidan and Schilling, 2004; Hall, 2015). Importantly, 70% of human genes have at least one ortholog in zebrafish and medaka genomes (Kasahara et al., 2007; Sasaki and Shimizu, 2008; Howe et al., 2013). In addition, the amenability of this two fish models to genetic manipulation/editing, e.g., through CRISPR/cas9 technology (Hwang et al., 2013; Ansai and Kinoshita, 2014; Kirchmaier et al., 2015; Cornet et al., 2018) have favored the development of several mutant lines capable of modeling human skeletal disorders. Other features such as a high number of offspring, a short generation time, an external development and translucent early life stages have potentiated the popularity of these two teleosts for bone studies. However, differences exist and those must be considered when using fish as models for OP studies. In this regard, osteocytes, which represent nearly 95% of all bone cells in mammals (Franz-Odendaal et al., 2006) and have been linked to bone loss in OP patients (Busse et al., 2010), are not present in all life stages and/or skeletal elements of teleosts (Witten and Huysseune, 2009; Apschner et al., 2011). In medaka, bone is totally devoid of osteocytes, while osteocytes are absent from zebrafish bone during early development and in exoskeletal structures such as scales and fins rays. Despite the absence of osteocytes, teleost bone is still subjected to resorption and remodeling (Witten and Huysseune, 2009) and is still responsive to mechanical loading (Suniaga et al., 2018; Ofer et al., 2019). Osteoclasts, the bone resorbing cells, are also slightly different. Large multinucleated cells in mammals, they are often small mononucleated cells in teleosts; however, resorbing activity is maintained (although lower in teleosts) and molecular pathways are conserved (Witten and Huysseune, 2009). It is worth to note that teleost fins and scales are dermal skeletal elements with no corresponding structures in mammals as they were largely lost during terrestrial evolution and replaced by non-calcified hair and feathers or keratin derived scales and horns; thus care should be taken when comparing results collected from these systems with those from human trabecular or compact bone.

## Glucocorticoid Induced Osteoporosis

Glucocorticoids (GCs) are potent immunosuppressive drugs used to treat inflammatory diseases; however, long-term therapies lead to complex adverse effects, including secondary osteoporosis. In humans, GCs impact bone metabolism by decreasing bone formation and vascularization and increasing bone resorption (reviewed in Chotiyarnwong and McCloskey, 2020). In conditions of glucocorticoid-induced osteoporosis (GIOP), osteoblasts experience increased apoptosis and have their activity and number reduced. Additionally, osteoclast proliferation, lifespan, and activity are increased in the initial phase of GIOP. Longer treatments, however, impair osteoclast formation and function (Buehring et al., 2013). Much remains to be elucidated on the mechanisms underlying osteoporosis and, today, no effective treatment for GIOP has been developed. Mammalian models mimicking GIOP (reviewed in Wood et al., 2018) have been preferentially used to uncover important pathophysiological aspects of the disease, although small teleost fish, such as zebrafish and medaka, were shown to respond in a similar way to GC treatment, confirming the evolutionary conservation of key players and pathways in GC metabolism, and represent a valuable option for the study of GIOP (Table 1). Barrett and colleagues demonstrated that tissue ossification was reduced in zebrafish larvae exposed to prednisolone and that phenotype could be partially rescued upon treatment with bisphosphonates (Barrett et al., 2006), a key similarity to human GIOP (Hayat and Magrey, 2020). Still in zebrafish larvae, resveratrol was shown to counteract the OP phenotype induced by dexamethasone (Luo et al., 2019), as described for mammals (Tou, 2015). Further studies aiming at validating the suitability of the fish model have evidenced an effect of prednisolone on bone homeostasis, through the altered expression of genes (i) involved in osteoblast (*entpd5a*) and osteoclast (*acp5a* and *sost*) differentiation, (ii) crucial to extracellular matrix (ECM) mineralization (*mmp9* and *mmp13*), and (iii) involved in pathways critical to osteoblast and osteoclast signaling (Huo et al., 2018). Adult zebrafish models have also been used to study GIOP. Because scales can be harvested from adult zebrafish with little or no stress and have the capacity to fully regenerate in 2 weeks, they are commonly used for *in vivo* and *ex vivo* bone research. Treatment of zebrafish with prednisolone during scale regeneration was shown to induce an osteoporotic phenotype in regenerates, due to an imbalanced bone formation. Scales of OP fish displayed an enhanced osteoclast activity, an increased matrix resorption and induced osteoporotic gene-expression profile in osteoblasts and osteoclasts (de Vrieze et al., 2014). It was additionally shown that prednisolone-treated zebrafish exhibited increased resorption lacunae and lower osteoblastic function, evidenced by decreased alkaline phosphatase (ALP) activity, and that concomitant treatment with alendronate restored ALP activity and significantly decreased tartrate-resistant acid phosphatase (TRAP) activity, a proxy for osteoclast function (Pasqualetti et al., 2015). In scales of male zebrafish, age was shown to potentiate the effect of prednisolone treatment, further increasing TRAP activity, *tnfrsf1b* expression, and the extent of the resorption lacunae (Carnovali et al., 2020a). Bony rays of zebrafish caudal fin were also found to be a suitable and reliable model to study the elemental, structural and mechanical characteristics of bones affected by anti- and pro-mineralogenic compounds. Exposure of zebrafish to prednisolone during fin ray *de novo* formation delayed bone growth and impaired bone regeneration by impacting on the number, activity and differentiation of osteoblasts and osteoclasts, but also immune cells (Geurtzen et al., 2017). Moreover, prednisolone altered the expression of several ECM related genes and continued exposure deteriorated cellular trafficking in the regenerating fin thus interfered with epithelial and bone tissue restoration upon amputation (Schmidt et al., 2019). Again, alendronate was able to prevent prednisolone effects on fin ray mineralization and calcium/phosphorous levels, and restored bone biomechanical properties (Bohns et al., 2020). There is, however, a striking difference between humans and zebrafish, when it comes to vertebrae, as adult zebrafish vertebrae are not affected by GC-induced bone loss. This could be partially explained by the low rates of osteoblast proliferation as GC treatment seems to have greater impact on bone tissues undergoing active osteoblast proliferation (Geurtzen et al., 2017).

**TABLE 1:** List of teleost models and respective assays to mimic mammalian osteopenic/osteoporotic phenotypes.

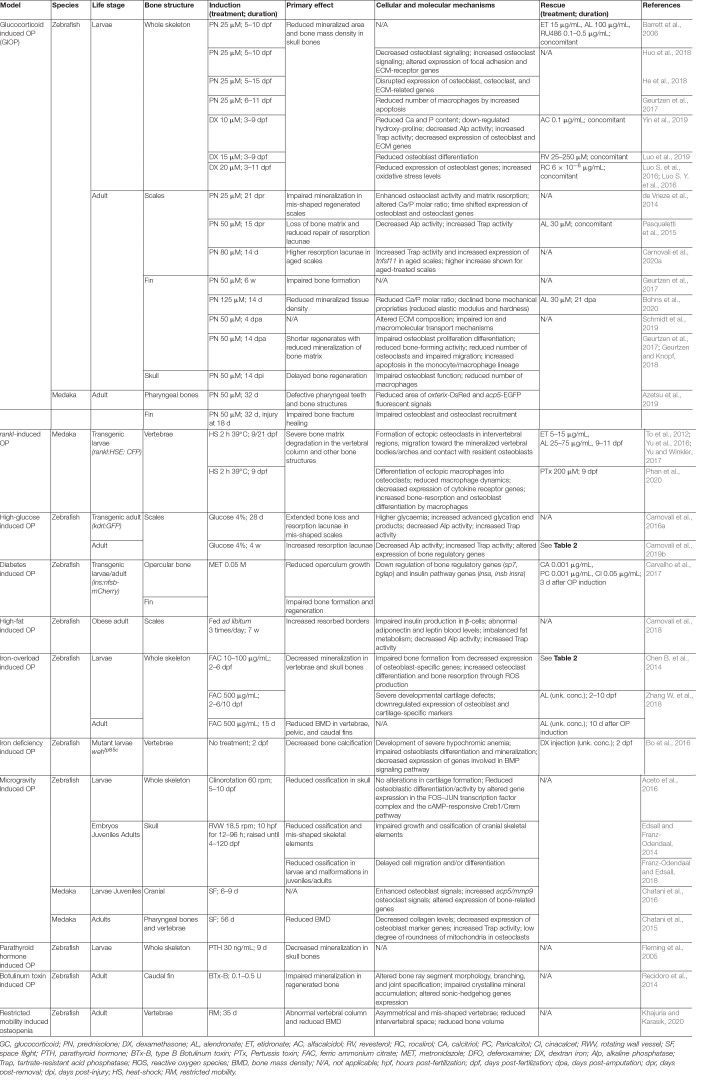

**GC, glucocorticoid; PN, prednisolone; DX, dexamethasone; AL, alendronate; ET, etidronate; AC, alfacalcidol; RV, revesterol; RC, rocalirol; CA, calcitriol; PC, Paricalcitol; CI, cinacalcet; RWV, rotating wall vessel; SF, space flight; PTH, parathyroid hormone; BTx-B, type B Botulinum toxin; PTx, Pertussis toxin; FAC, ferric ammonium citrate; MET, metronidazole; DFO, deferoxamine; DX, dextran iron; Alp, alkaline phosphatase; Trap, tartrate-resistant acid phosphatase; ROS, reactive oxygen species; BMD, bone mass density; N/A, not applicable; hpf, hours post-fertilization; dpf, days post-fertilization; dpa, days post-amputation; dpr, days post-removal; dpi, days post-injury; HS, heat-shock; RM, restricted mobility.**

## High Glucose and High Fat Induced Osteoporosis

Diabetes is a chronic metabolic disease that is growing worldwide (Lin et al., 2020). It has a negative impact on many tissues/organs, including the skeleton, where it may trigger or potentiate the development of osteoporosis (Cipriani et al., 2020). Together, diabetes and osteoporosis are responsible for severe clinical complications that impact on patients’ quality of life and impose an important economic burden to health systems (Leung et al., 2017). Epidemiologic studies have also evidenced positive associations between obesity and bone health (Zhao et al., 2007) and an interplay between type 2 diabetes and overweight (reviewed in Al-Goblan et al., 2014). Although recent studies have evidenced interactions between bone and glucose or fat metabolism, much remains to be understood concerning the molecular players and their interaction with genetics and environment. Because it has become a widespread model to investigate metabolic diseases such as diabetes and obesity (Zang et al., 2018; Carnovali et al., 2019b; and references therein) and skeletal pathologies (Tonelli et al., 2020 and references therein), zebrafish is therefore a model of choice to investigate the interplay between hyperglycemia/obesity and bone, under pathological conditions (Table 1). In humans, chronic hyperglycemia is known to negatively affect bone homeostasis resulting in increased bone fragility, reduced mechanical strength and increased fracture risk due to impaired ECM formation and bone cells function (Cipriani et al., 2020). In a zebrafish model of diabetes [*Tg(ins:nfsb-mCherry)*], it was shown that both larval osteogenesis and the regenerative capacity of caudal fin rays was impaired under hyperglycemia, and that this condition can be rescued by treatment with the vitamin D analog (paricalcitol) and a calcimimetic (cinacalcet) (Carvalho et al., 2017). Additionally, studies using adult zebrafish exposed to glucose have revealed a decrease in scale matrix mineralization, the presence of bone resorption lacunae associated with an intense osteoclast activity and altered expression of bone regulatory genes (Carnovali et al., 2016a, 2019b), similarly to what has been recently reported in bones of diabetic rodents (An et al., 2019) and humans (Karner and Long, 2018). The presence of bone resorption lacunae associated with intense osteoclastic TRAP activity and decreased osteoclastic ALP activity was also observed in the scales of adult zebrafish fed a high-fat diet and showing signs of obesity (Carnovali et al., 2018).

## Iron Overload Induced Osteoporosis

Iron plays an essential role in human physiology as a co-factor in biochemical reactions involved in critical metabolic functions such as oxygen transport and storage, respiratory chain complexes and DNA replication (Frazer and Anderson, 2014; Bogdan et al., 2016). Aging has been associated with iron accumulation in different tissues and the production of reactive oxygen species (ROS; reviewed in Picca et al., 2019), which contribute to promote or potentiate the severity of age associated pathologies such as osteoporosis and osteoarthritis (Liu et al., 2006). Excess of iron also promotes osteoclast differentiation in mouse and increased bone resorption (Camacho et al., 2016; Simão et al., 2018, 2019). In zebrafish larvae, iron overload inhibited bone formation and decreased expression of osteoblast marker genes, allegedly due to an increased ROS production and the associated oxidative stress (Chen B. et al., 2014). Larvae of the zebrafish mutant expressing a defective Ferroportin 1, a cellular iron exporter required for iron cycling, exhibited, in addition to severe hypochromic anemia, clear defects in bone formation, including a reduced number of calcified vertebrae and the abnormal expression of osteoblastic genes (Bo et al., 2016). High-iron stress was also used to promote an osteoporosis-like phenotype in zebrafish (Zhang W. et al., 2018; Table 1). Both larvae and adult fish showed a significant decrease in bone mineralization and severe developmental defects in cartilage formation. Interestingly, defective osteogenesis was significantly rescued using alendronate, a drug known to be effective against human osteoporosis by targeting the Bmp signaling pathway and promoting osteoblast differentiation (Zhang S. et al., 2018).

## RANKL-Overexpression Induced Osteoporosis

Receptor activator of NF-κB ligand (RANKL) and its receptor RANK play a pivotal role in the differentiation of cells of the monocyte/macrophage lineage into functional osteoclasts and are therefore critical regulators of osteoclast-mediated bone resorption (reviewed by Walsh and Choi, 2014). Many scientists believe that RANK/RANKL system is a target of choice for therapies aiming at treating disorders associated with altered bone remodeling, such as osteoporosis (reviewed by Matsumoto and Endo, 2020). Clinical studies evaluating the suitability of RANKL inhibition to treat postmenopausal osteoporotic women have shown that denosumab, a human monoclonal antibody that specifically neutralizes RANKL, significantly reduced the incidence of vertebra and hip fractures, and improved patients prognosis (reviewed by Dempster et al., 2012). Interestingly, the critical role of RANKL in osteoclastogenesis has been conserved throughout evolution, as increased expression of *rankl* triggered an osteoporotic bone phenotype in medaka (To et al., 2012; Table 1). In *rankl* transgenic fish, active osteoclasts were formed ectopically and promoted bone resorption in mineralized arches and vertebral bodies, in a way that resembles findings in human osteoporosis. Besides phenotypical similarities, *rankl*-induced ectopic osteoclasts were shown to derive from a subset of macrophages that migrated and accumulated into the vertebral column, and then interacted with osteoblast progenitors before differentiating into bone resorbing osteoclasts (Phan et al., 2020). Transcriptome profiling of Rankl-activated macrophages revealed the differential expression of several osteoclast markers, e.g., *trap*, *ctsk*, *siglec15*, *nfatc1*, *tgfb1*, and *dap12*, a situation also observed in mammals (Phan et al., 2020 and references therein). Another feature common to both fish and mammalian OP models, is the similar response to genetic or chemical inhibition of *tnf*α, which improved bone loss in both models, by reducing ectopic osteoclast differentiation in a selective manner (Phan et al., 2020). The rescue of OP phenotype by bisphosphonates was also evaluated in adult medaka, and both alendronate and etidronate inhibited osteoclast activity in a dose-dependent manner upon *rankl* induction, thus contributing to the maintenance of bone homeostasis and integrity. Rescue by bisphosphonates was also reported in osteoporotic larvae (Yu et al., 2016; Yu and Winkler, 2017). The morphological changes that occurred in fish osteoclasts during treatment, e.g., formation of aggregates and fusion into giant multinucleated cells, were remarkably similar to those seen in patients after long-term treatment with bisphosphonates (Jobke et al., 2014) and is another indication of conserved mechanisms between teleosts and humans.

## Microgravity Induced Osteoporosis

Microgravity experienced by astronauts during spaceflights reduces the mechanical loading on bones and triggers bone loss, skeletal deterioration and imbalanced calcium metabolism (Keller and Strauss, 1993; Turner, 2000). To better understand these effects, animal models were sent to space or submitted to microgravity, among them the zebrafish and the medaka (Moorman et al., 1999; Shimada et al., 2005; Aceto et al., 2016). Larval zebrafish responded to microgravity by reducing otolith (mineralized rods of the inner ear) development but also by altering the expression of genes important for the development of the notochord, which participates in the patterning and formation of the vertebral column (Moorman et al., 1999). Simulated microgravity (SMG) by 2D-clinorotation for 5 days significantly decreased bone formation in zebrafish larvae and altered the expression of genes involved in bone metabolism and formation but also genes associated with disorders in connective tissue, skeleton and muscle (Aceto et al., 2016; Table 1). Zebrafish embryos exposed to SMG at key developmental stages developed long term skeletal effects such as a reduced ossification and skeletal malformations (Edsall and Franz-Odendaal, 2014). Adult medaka and transgenic larvae sent to the International Space Station exhibited a decreased osteoblastic activity, an increased osteoclastic activity and a reduced BMD, i.e., signs of microgravity-induced osteoporosis (Chatani et al., 2015, 2016). Promoter activity and expression of several osteoclast marker genes (e.g., *trap*, *ctsk*, and *mmp9*) were also stimulated during spaceflight, indicating enhanced osteoclastic activity. Interestingly, GC-related genes (e.g., *fkbp5* and *ddit4*), known to contribute to osteoblast and osteoclast genesis, were also upregulated. In contrast, exposure of zebrafish to hypergravity increased bone formation by modifying the expression profile of important bone markers (Aceto et al., 2015) and altered cartilage properties (Lawrence et al., 2021). Beside the possibility to identify new genes involved in bone formation and response to mechanical loading, hypergravity may also hold some potential for research on osteoporosis.

## Osteopenia Models

Osteopenia is characterized by a mild reduction of BMD and often leads to osteoporosis if not detected and corrected in due course. Fish models with adaptive osteopenia (a low BMD condition that evolved in some fish as an adaptive mechanism) or induced osteopenia (a low BMD condition that is induced in fish placed in a particular situation or exposed to compounds) have been described and are detailed below. Antarctic fish of the notothenioid family, in particular the channichthyids or icefish, have evolved a weakly ossified skeleton in response to their habitat and the need to increase their buoyancy in the water column for feeding (Albertson et al., 2009; Eastman et al., 2014). The reduction of the BMD in icefish, favored throughout millions of years of evolution, exhibits characteristics similar to human osteopenia and could hold important clues for the better understanding of disease mechanisms. However, the difficulty to grow and study icefish in captivity has hindered their use as model organisms and their wide distribution in research laboratories. Reduced BMD conditions that could mimic osteopenia phenotype are also found in fish exposed to fasting and intensive swimming, situations that frequently occur during the migration of fishes such as eels and salmonids (Kacem and Meunier, 2003; Rolvien et al., 2016). Under these conditions of severe physiological stress, bone demineralization occurs through a mechanism that may involve the action of glucocorticoids and the activation of the neuroendocrine axis (Suarez-Bregua et al., 2018). In this regard, a continuous exposure of zebrafish larvae to parathyroid hormone resulted in a marked decrease of bone mineralization after 6 days of treatment (Fleming et al., 2005). Under appropriate conditions of stress (e.g., fasting), fish could develop an osteoporotic phenotype resembling human disease that could contribute to a better understanding of bone demineralization (Schartl, 2014). Restricted mobility, i.e., limited swimming activity, was also used to induce osteopenia in adult zebrafish (Table 1). Upon movement restriction and decreased mechanical loading, the shape of the vertebrae and the thickness of the intervertebral space were altered, while bone volume and density were reduced (Khajuria and Karasik, 2020). Following a mechanism that may be related to reduced mechanical loading but also to disrupted nerve function, the transient paralysis of zebrafish muscle upon Botulinum toxin injection also impaired intramembranous ossification during caudal fin regeneration (Recidoro et al., 2014).

## Fracture Osteoporosis Models

Several teleost skeletal structures—skull, fin and scales—have been used to better understand the fracture-healing processes and identify therapeutic agents (Sousa et al., 2012; Geurtzen et al., 2014; Kobayashi-Sun et al., 2020). Although osteoblasts were found to play a major role in fracture healing in both mammals and teleosts, their origin seems to be different in these two groups of vertebrates. In zebrafish, mature osteoblasts will dedifferentiate in more proliferative and active cells during the repair of fin fractures and skull injuries, a mechanism also observed during fin regeneration (Geurtzen et al., 2014), while a new set of osteoblasts is formed in mammals from mesenchymal stem cells to contribute to the repair of bone injuries (Park et al., 2012). Also conserved throughout evolution, the detrimental effect of GCs on fracture healing was observed in mouse (Liu Y. Z. et al., 2018) and in teleosts, delaying skull repair in zebrafish (Geurtzen et al., 2017) and fin repair in medaka (Azetsu et al., 2019), probably due to impaired osteoblast recruitment. A recent study using zebrafish scale fracture model, reported that osteoclast differentiation during fracture healing is mediated by extracellular vesicles released by osteoblasts (Kobayashi-Sun et al., 2020). Although the conservation of this mechanism in mammals remains to be confirmed, data collected in teleosts shed some light on a possible alternative process toward bone repair.

## Applications

Validated teleost OP models (Table 1) have been used in a wide range of applications. The literature associated with these applications has been expanding rapidly, with many publications exploring disease mechanisms and others discovering novel therapeutic targets and compounds to treat disease symptoms. Most of these studies used zebrafish GIOP model, probably because it is a model that can be easily implemented in any laboratory, but also because it addresses the most prevalent type of secondary osteoporosis (Compston, 2018). However, the importance of the other teleost OP models was not disregarded, as they have allowed to unveil many conserved aspects of OP pathology. Below are examples of the most popular applications using teleost OP models (see also Table 2).

**TABLE 2:** Fields of application of teleost OP models.

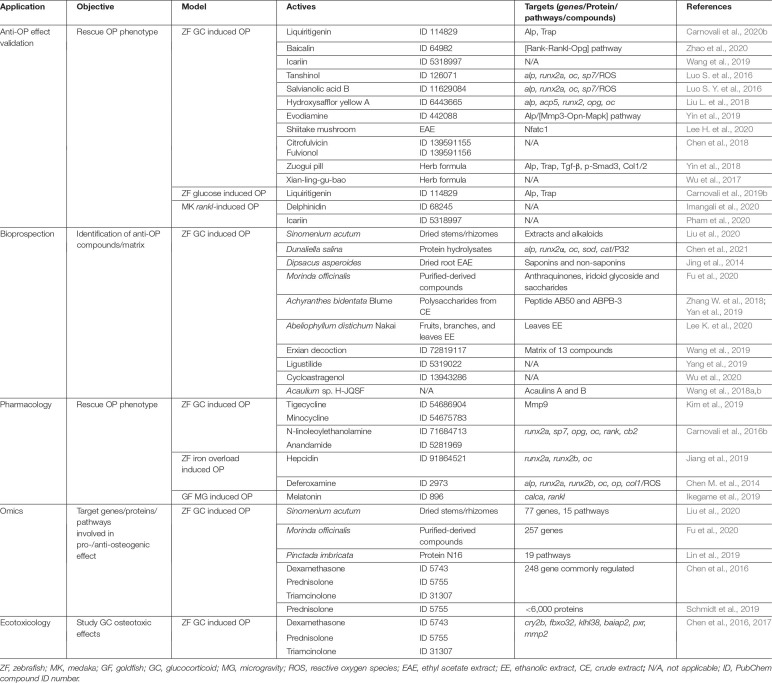

**ZF, zebrafish; MK, medaka; GF, goldfish; GC, glucocorticoid; MG, microgravity; ROS, reactive oxygen species; EAE, ethyl acetate extract; EE, ethanolic extract, CE, crude extract****;****N/A, not applicable; ID, PubChem compound ID number.**

### Anti-OP Compound Validation

Many extracts and compounds derived from plants and herbs are commonly used worldwide as nutraceuticals and largely explored in traditional Chinese medicine (TCM), because of their antioxidant, anti-inflammatory, anti-tumoral, neuro-protective, anti-diabetic, anti-angiogenic and anti-atherosclerotic proprieties (Che et al., 2016). Several of these natural extracts/compounds have also been claimed to have anti-resorptive and bone anabolic activities, but strong scientific data was missing to validate these claims. Glucocorticoid and glucose-induced OP (zebrafish) and *rankl*-induced OP (medaka) are the disease models that have been used to confirm their osteoactivity and identify their mechanisms of action. For example, anti-resorptive properties of the flavonoids liquiritigenin (Carnovali et al., 2019b, 2020b), baicalin (Zhao et al., 2020), icariin (Wang et al., 2019; Pham et al., 2020), and the pigment delphinidin (Imangali et al., 2020) was shown to occur through impaired osteoclast activity and reduced bone resorption. Other phenolic compounds, such as tanshinol (Luo S. et al., 2016) and salvianolic acid B (Luo S. Y. et al., 2016), were shown to have bone anabolic properties, and hydroxysafflor yellow A was found to have both bone anabolic and anti-resorptive properties (Liu L. et al., 2018). The ability of alkaloids such as evodiamine (Yin et al., 2019) to trigger bone remodeling and the capacity of fungal extracts—e.g., from shiitake mushroom (Lee H. et al., 2020) and from *Penicillium velutinum* (Chen et al., 2018)—to successfully rescue OP phenotype was successfully demonstrated in the zebrafish GIOP model. Osteoactivity of the TMC formulations Xian-ling-gu-bao (Wu et al., 2017) and Zuogui pill (Yin et al., 2018), commonly used in Asiatic countries to treat primary OP, was also addressed in the zebrafish GIOP model, unveiling their potential to also counteract secondary forms of OP.

### Bioprospection

It is clear from the previous paragraph that natural extracts contain promising osteoactive compounds that may be used to develop next-generation drugs to treat osteoporosis. Because they bring many technical advantages into the cumbersome screening process, e.g., they allow larger and quicker screening assays, teleost OP models, in particular the zebrafish GIOP model, have been favored as first line screening assays and several osteogenic fractions and compounds derived from plants and fungi have already been identified (Niu et al., 2015; Lee K. et al., 2020). Holistic approaches coupling network pharmacology modulation and zebrafish GIOP model as a screening platform to test the efficacy of identified matrixes were also successfully implemented. These allowed, for example, to isolate osteogenic enriched extracts derived from Erxian decoction (Wang et al., 2019), osteogenic alkaloids extracts from *S. acutum* (Liu et al., 2020), but also osteogenic peptides, polysaccharides and other anabolic compounds from microalgae (Chen et al., 2021) and plants (Zhang S. et al., 2018; Yan et al., 2019; Fu et al., 2020). Teleost OP models were also used to screen natural compound libraries and unveil new activities for old compounds (Yang et al., 2019; Wu et al., 2020).

### Pharmacology

Pharmaceuticals unrelated to OP treatment have been found to have osteoactive properties in teleost OP models. For example, tetracycline analogs—a well know class of antibiotics—were shown in zebrafish GIOP model to prevent OP phenotype (Kim et al., 2019). Long-chain fatty acid amides were found to stimulate bone formation in the same model (Carnovali et al., 2016b). Hepcidin, a protein involved in the regulation of iron levels in blood, and deferoxamine, a drug that binds circulating iron, were able to rescue iron overload induced bone loss and to increase bone formation in zebrafish (Chen B. et al., 2014; Jiang et al., 2019). Another example that illustrates the diversity of the applications for teleost OP systems was the use of goldfish scales to confirm the ability of melatonin to prevent bone loss in a microgravity environment. Melatonin, previously shown to improve bone mass in postmenopausal osteopenic women (Amstrup et al., 2015), was effective in suppressing goldfish osteoclast activity during a space flight experiment, indicating that it could be a promising treatment for bone loss experienced by astronauts (Ikegame et al., 2019).

### Omics

The global analysis of gene expression and protein production is an important approach to unveil the genes, pathways and proteins involved in disease development and treatment, and to identify putative therapeutic targets. In this sense, transcriptomic and proteomic analyses were applied to zebrafish exposed to GCs followed by treatment with different bone anabolic compounds, resulting in the identification of several possible therapeutic targets, thus potentially opening new routes for OP treatment (Chen et al., 2017; Lin et al., 2019; Schmidt et al., 2019; Fu et al., 2020; Liu et al., 2020).

### Ecotoxicology

A less obvious, but not less important application of teleost OP models, is their use as ecotoxicology tools. Synthetic GCs (e.g., prednisolone and dexamethasone) have been detected in the aquatic environment and the biological significance of this contamination has raised serious concerns. Zebrafish was used to confirm that environmental concentrations of GCs have the capacity to alter the expression of genes involved in GCs associated pathways and may potentiate disease mechanism (Chen et al., 2016, 2017), raising public awareness to a problem that may also impact on human (bone) health.

## Complementary Approaches Using Osteoporosis Genetically Modified Models

In addition to the above-mentioned models, morphants, stable knockdown and mutant zebrafish lines displaying phenotypic features that partially mimic OP are available (they have been extensively discussed by Laizé et al., 2014; Bergen et al., 2019; Carnovali et al., 2019a; Tonelli et al., 2020; Dietrich et al., 2021). These models are valuable tools to study OP-related genes and mechanisms associated with low BMD and fragile bones, and can be used in compound screening assays. Relevant examples in zebrafish are the *atp6v1h* mutant, which exhibits a severe reduction in the number of mature osteoblasts and an increase in *mmp9* and *mmp13* expression (Zhang et al., 2017), the *bmp1 frilly fins* (Asharani et al., 2012) and *col1a1 chihuahua* (Fisher et al., 2003) mutants, which show general defects in bone growth that lead to weak bones prone to fractures, the *gpr137ba* mutant (Urso et al., 2019) characterized by an increased bone resorption, the *gba1* morphant (Zancan et al., 2015) characterized by impaired osteoblast differentiation and reduced bone mineralization, and the *sp7* mutant (Kague et al., 2016) presenting a delayed bone maturation and unregulated bone formation. On a different aspect, the use of teleost systems to study gene-specific effects upon pharmacological manipulation holds a great potential for the screening of anti-OP compounds. For example, SOST is a protein secreted in human by the osteocytes (Delgado-Calle et al., 2017), and its mutated form was associated to high bone mass (HBM) disorders (Balemans et al., 2001). By inhibiting SOST binding to LRP5/6, the monoclonal antibody romosozumab could stimulate bone deposition and improve OP patients’ condition (McClung, 2018). Interestingly, bone remodeling in zebrafish and medaka is also mediated by *sost*, although not expressed by osteocytes, and the down-regulation of *sost* expression resulted in more bone deposited (Ofer et al., 2019). Thus teleosts could be used for large screening assays aiming at identifying compound that could modulate *sost* expression toward an increased bone deposition. A similar example is associated with the modulation of *smad9* expression in zebrafish (Gregson et al., 2020; McDonald et al., 2021). These two examples highlight the evolutionary conservation of skeletal mechanisms from fish to human and reinforce the suitability of teleost models for target-based screening assays. Data from genome wide association studies (GWAS) and human epidemiological analysis of skeletal diseases represent a promising source of new targets to be validated and further characterized in teleosts.

## Conclusion

The high degree of conservation of the mechanisms involved in skeletogenesis between fish and mammals allows to recapitulate, with a notable similarity, OP phenotypes in zebrafish and medaka, two small teleosts that can be incorporated in high-throughput screening pipelines and thus hold great promises for the discovery of anti-OP drugs. Among the different systems described in this review, glucocorticoid-induced OP in zebrafish is by far the most popular system, being easily operable in most laboratories and necessitating only wild-type zebrafish lines. Although the transgenic medaka line overexpressing *rankl* is meant to gain more importance in the future as it represents the most suited OP model developed so far, particular attention should be given to fish HBM models currently being developed as they provide an interesting alternative approach to classical OP models that may prove to be more fruitful. The positive impact of fish OP models on the identification of compounds with anti-OP activity is already perceptible and is likely to gain momentum in the next decade. Due to their enormous potential, it is also possible that we see fish OP models becoming more prominent models for pre-clinical OP studies.

## Author Contributions

JR drafted the tables. All authors contributed to the literature research, to the writing, editing of the manuscript, and approved the submitted version.

## Conflict of Interest

The authors declare that the research was conducted in the absence of any commercial or financial relationships that could be construed as a potential conflict of interest.
